# Epidermal Growth Factor Receptor Inhibition Reduces Angiogenesis via Hypoxia-Inducible Factor-1α and Notch1 in Head Neck Squamous Cell Carcinoma

**DOI:** 10.1371/journal.pone.0119723

**Published:** 2015-02-27

**Authors:** Wei-Ming Wang, Zhi-Li Zhao, Si-Rui Ma, Guang-Tao Yu, Bing Liu, Lu Zhang, Wen-Feng Zhang, Ashok B. Kulkarni, Zhi-Jun Sun, Yi-Fang Zhao

**Affiliations:** 1 The State Key Laboratory Breeding Base of Basic Science of Stomatology & Key Laboratory of Oral Biomedicine Ministry of Education, Wuhan University, Wuhan, China; 2 Department of Oral and Maxillofacial-Head and Neck Oncology, School and Hospital of Stomatology, Wuhan University, Wuhan, China; 3 Functional Genomics Section, Laboratory of Cell and Developmental Biology, National Institute of Dental and Craniofacial Research, National Institutes of Health, Bethesda, Maryland, United States of America; Winship Cancer Institute of Emory University, UNITED STATES

## Abstract

Angiogenesis, a marker of cancer development, affects response to radiotherapy sensibility. This preclinical study aims to understand the receptor tyrosine kinase-mediated angiogenesis in head neck squamous cell carcinoma (HNSCC). The receptor tyrosine kinase activity in a transgenic mouse model of HNSCC was assessed. The anti-tumorigenetic and anti-angiogenetic effects of cetuximab-induced epidermal growth factor receptor (EGFR) inhibition were investigated in xenograft and transgenic mouse models of HNSCC. The signaling transduction of Notch1 and hypoxia-inducible factor-1α (HIF-1α) was also analyzed. EGFR was overexpressed and activated in the *Tgfbr1/Pten* deletion (2cKO) mouse model of HNSCC. Cetuximab significantly delayed tumor onset by reducing tumor angiogenesis. This drug exerted similar effects on heterotopic xenograft tumors. In the human HNSCC tissue array, increased EGFR expression correlated with increased HIF-1α and micro vessel density. Cetuximab inhibited tumor-induced angiogenesis *in vitro* and *in vivo* by significantly downregulating HIF-1α and Notch1. EGFR is involved in the tumor angiogenesis of HNSCC via the HIF-1α and Notch1 pathways. Therefore, targeting EGFR by suppressing hypoxia- and Notch-induced angiogenesis may benefit HNSCC therapy.

## Introduction

Head and neck squamous cell carcinoma (HNSCC) ranks as the sixth most frequent cancer worldwide with approximately 500,000 new cases per year worldwide[1]. Previous studies have established that risk factors, such as alcohol drinking, smoking, and human papilloma virus infection, contribute to the development of this fatal disease [2]. However, the five-year survival rate of HNSCC patients remains relatively unchanged at 40% to 50% during the past three decades [3]. Advanced-stage HNSCC patients have poor prognosis and often need both chemotherapy and radiotherapy [4]. However, only 30% of advanced-stage HNSCC patients survive for more than 5 years. Important factors that contribute to this scenario include the relative hypoxic and angiogenic conditions of high tumor burden in HNSCC. These conditions promote the stemness of cancer stem cells with both local and distant metastatic potentials [5].

Emerging basic, preclinical, and clinical findings indicated that epidermal growth factor receptor (EGFR)-mediated aberrant signaling transduction is crucial in HNSCC tumorigenesis and progression [6]. EGFR has been observed in 70% to 100% of all HNSCC lesions [7]. The high phosphorylation status of EGFR is frequently correlated with poor prognosis [8]. Activated EGF/EGFR pathway may promote cell proliferation, differentiation, angiogenesis, and anti-apoptosis in HNSCC tumorigenesis and progression through the phosphoinositide-3-kinase (PI3K)/Akt, ras/raf/extracellular regulated protein (Erk), and signal transducer and activator of transcription pathways [9, 10]. Cetuximab is a chimeric IgG1 monoclonal antibody that is currently licensed for the treatment of HNSCC patients [11, 12]. This drug is used alone or in combination with chemotherapy as the first and second lines of treatment for advanced-stages patients [13]. Hypoxia-inducible factor-1α (HIF-1α) is a principal molecular mediator for tumor angiogenesis, and Notch pathway dysregulation is a leading genetic instability in HNSCC [14–16]. Previous reports suggested that the interaction between HIF-1α and Notch1 can influence tumor angiogenesis [17]. However, the mechanism by which the interaction between EGFR and HIF-1α or Notch1 in HNSCC regulates angiogenesis and tumorigenesis has yet to be elucidated.

In our previous studies, we established that *Tgfbr1* and *Pten* conditional knock out (2cKO) mice demonstrate spontaneous fast HNSCC tumorigenesis with 100% penetration [18]. HNSCC mice are highly angiogenic as compared with *Pten* knock out HNSCC mice [19]. The present study shows that the overexpression and high phosphorylation of EGFR are crucial for the tumorigenesis of transgenic mouse models with combined *Tgfbr1* and *Pten* loss. Furthermore, the cetuximab-induced inhibition of EGFR repressed tumor burden in xenograft HNSCC models. Chemopreventive treatment with cetuximab delays HNSCC onset in *Tgfbr1/Pten* 2cKO mice and reduced HIF-1α- and Notch1-mediated angiogenesis. EGFR overexpression was correlated with HIF-1α and micro vessel density (MVD) in HNSCC clinical specimens. Thus, HIF-1α- and Notch1-mediated angiogenesis may be important for EGFR activation and may partially contribute to EGFR inhibitor sensitivity.

## Materials and Methods

### Chemicals and reagents

All chemicals and reagents were obtained from Sigma-Aldrich (St. Louis, MO, USA), unless indicated. Antibodies against EGFR, p-EGFR^Tyr1068^, HIF-1α, and Notch1, Notch1 intracellular domain (NICD), Hes1, VEGF, Histone H3 were obtained from Cell Signaling Technologies (Danvers, MA, USA), CD31 were obtained from BD Pharmingen (NJ, USA). Cetuximab was purchased from Merck (Darmstadt, Germany). *N*-[*N*-(3,5-difluorophenacetyl-l-alanyl)]-*S*-phenylglycine *t*-butyl ester (DAPT, γ-secretase inhibitor which inhibited cleavage of Notch1) was obtained from Sigma-Aldrich (St. Louis, MO, USA).

### Cell culture, conditional medium collection and *in vitro* migration assay

The CAL27 cell line was purchased from ATCC and cultured in Dulbecco’s modified eagle medium (DMEM) supplemented with 10% FBS as previous described [20], in a humidified atmosphere of 95% air, 5% CO_2_ at 37°C. CAL27 cells were serum-deprived for 12h and then treated with or without cetuximab (10 μg/ml) or DAPT (20 μM) in for indicated time (12h) in Anoxomat chambers (Mart Microbiology, Lichtenvoorde, the Netherlands) with appropriate oxygen concentrations for hypoxia (1% O_2_) or normoxia (21% O_2_). The cells were washed by phosphate buffer solution (PBS) two times and continue grow in serum-deprived endothelial basic medium (EBM, Lonza, Walkersville, MD, USA) medium for another 24h, and the cleared supernatants were collected as conditional medium (CM) and stored at -80°C. Pooled human umbilical vein endothelial cells (HUVECs) were purchased from Lonza and cultured as previous described [19]. *In vitro* wound healing assay and Boyden chamber transwell migration assay and tube formation assay of HUVECs were performed as previous described [19] with detail in Supplementary Material and Methods in S1 File.

### RNA interference

RNA interference were performed as previous described [20].Briefly, CAL 27 cells were seeded in 6cm culture dishes and allowed to grown to 80% confluence, transfected with TGFBR1 siRNA or/and PTEN siRNA with Hiperfect transfection reagent (Qiagen) according to the manufacturer’s instruction. The knock down efficiency with at least 84% decrease of TGFBR1 or PTEN protein at a indicated time (24h) were confirmed by western blot as previous described [20].The expression of EGFR, p-EGFR^Tyr1068^ after the transfection was confirmed by Western blots.

### Cell immunofluorescence and confocal microscopy

Immunofluorescence were performed as previous described [19] and detail described in Supplementary Material and Methods in S1 File. Cells immunofluorescence was photographed by microscopy (CLSM-310, Zeiss, Germany).

### Nuclear/cytosolic fractionation

The nuclear/cytosolic fractionation of CAL27 cells was extracted using a Nuclear-Cytosol Extraction Kit (Applygen Technologies, Beijing, China) according to the manufacturer’s instructions. Briefly, CAL27 cells treated with or without cetuximab were collected by centrifugation and resuspended in cytosol extraction buffer A. After incubation on ice for 10 min, the cells were mixed with cytosol extraction buffer B and further incubated on ice for 1 min. The lysates were separated by centrifugation, and the supernatant (cytosol extract) was collected and transferred into a new tube. The pellet was washed with cytosol extraction buffer A, and resuspended in cold nuclear extraction buffer. After incubation at 4°C for 30 min with constant rotation, the suspension was centrifuged at 12,000 *g* at 4°C for 5 min to collect the nuclear extract in the supernatant fraction. The nuclear and cytoplasmic extracts were subjected to Western blots analysis.

### Establishment and cetuximab treatment of CAL27 heterotopic xenograft tumors model in nude mice

All animal studies include nude mice and transgenic mice were approved and supervised by Animal Care and Use Committee of Wuhan University and conducted in accordance with the NIH guidelines for the Care and Use of Laboratory Animals. Female athymic nude mice (18–20 g; 6–8weeks of age) were obtained from the Experimental Animal Center of Wuhan University in pressurized ventilated cage according to institutional regulations. Mice were housed in appropriate sterile filter-capped cages and with an inverse 12 h day-12 h night cycle. Lights were turned on at 8:30 am at 22 ± 1°C and 55 ± 5% humidity in the Experimental Animal Center of Wuhan University. All cages contained wood shavings, bedding and a cardboard tube for environmental enrichment. Animals fed and watered ad libitum.

For heterotopic xenograft, nude mice were injected subcutaneously with CAL27 cells (4×10^6^ in 0.2 ml of serum-free medium) in the flank when cells exponentially grow. After tumors were established, the mice were divided into two groups randomly, which were received cetuximab (10 mg/kg i.p. twice per week; n = 5) or normal saline (vehicle, 100ul i.p. 2/week; n = 5) infusion for 3 weeks. Tumor growth was determined by measuring the size of the tumors 3 times per week. The formula (width^2^×length)/2 was used to determine tumor volumes. All mice were monitored daily for abnormal behavior, e.g., inability to eat or drink, unable to run away when touched, no response to stimuli. There was no mice which was euthanized before the experimental endpoint. The maximum tumour sizes reached to 1.2 cm during the course of this assay. The mice were euthanized using CO_2_ and the tumors were harvested for the following immunohistochemical analysis and western blots analysis.

### Chemopreventive study on *Tgfbr1/Pten* combined conditional knockout (2cKO) mice

The squamous epithelial tissue specific and time inducible combined *Tgfbr1/Pten* knockout mice (*Tgfbr1/Pten* 2cKO, *K14-Cre* ^*ERtam*^; *Tgfbr1* ^flox/flox^; *Pten* ^flox/flox^) were maintained as previously described [18, 21]. The *Tgfbr1/Pten* 2cKO mice and their vehicles (*Tgfbr1* ^flox/flox^ *; Pten* ^flox/flox^) were from the same litter with mixed genetic background of C57BL/6; FVBN; CD1;129. Five day consequent tamoxifen oral gavage need to applied to knock out *Tgfbr1/Pten* in oral epithelial and head neck skin. The tamoxifen application procedure has been previously described [18, 21]. Only 4- to 8-week-old male and female *Tgfbr1/Pten* 2cKO mice were included in this study. For in chemopreventive assay, 2 weeks after the last dose of oral tamoxifen application of the *Tgfbr1/Pten* 2cKO mice were randomized into a vehicle group (100ul PBS. i.p. n = 5 mice) or a cetuximab group (10 mg/kg i.p. twice per week, n = 6 mice), based on our pilot study on the tumorigenesis and survival of 2cKO mice. All mice were monitored daily for abnormal behavior, e.g., inability to eat or drink, unable to run away when touched, no response to stimuli. There was no mice which was euthanized before the experimental endpoint. The maximum tumour sizes reached to 1.0 cm during the course of this assay. At the end of studies, mice were euthanized using CO_2_, tissues were harvest for histology immunohistochemical analysis and western blots analysis..

### Mouse phospho-Receptor Tyrosine Kinase (RTK) detection

For mouse phospho-RTK detection, we collected tissue of Tgfbr1/Pten 2cKO mouse tongue (n = 5), *Tgfbr1/Pten* 2cKO mouse tongue squamous cell carcinoma (n = 5), and their vehicles (*Tgfbr1* ^flox/flox^/*Pten* ^flox/flox^ tongue; n = 5) 6 weeks after the last oral tamoxifen dose. Antibody array was purchased from R&D system (proteome profiler mouse phospho-RTK array kit, ARY014). This array can detect the relative phosphorylation of 39 RTKs. Briefly, bovine serum albumin blocked the membrane containing immobilized phospho-RTK on a rocking platform at room temperature for 1 h. The membrane was then incubated with lysates of *Tgfbr1/Pten* 2cKO mouse tongue (n = 5), *Tgfbr1/Pten* 2cKO mouse tongue squamous cell carcinoma (n = 5), and their vehicles (Tgfbr1^flox/flox^/Pten^flox/flox^ tongue; n = 5) with Detection Antibody Cocktail overnight at 2°C to 8°C on a rocking platform. The membrane was incubated with horseradish peroxidase-conjugated secondary antibody (Pierce Chemical, Rockford, IL) and then with chemiluminescent detection reagent. The membrane was scanned, and pixel density was presented by quantifying the mean spot densities from two experiments. For western blot, we collected tissue of *Tgfbr1/Pten* 2cKO mouse tongue (n = 2), *Tgfbr1/Pten* 2cKO mouse tongue squamous cell carcinoma (n = 5), and their vehicles (*Tgfbr1* ^flox/flox^/*Pten* ^flox/flox^ tongue; n = 2).

### Human HNSCC tissues array

HN803 tissue arrays which contain 10 cases of normal tongue mucosa, 4 cases of lymph node metastasis and 57 confirmed cases of HNSCC were obtained from Biomax US (Rockville, MD, USA). The tissue array clinical data, including pathological classification and TNM classification were also provided by Biomax.

### Histology, immunohistochemistry and scoring system

Antibodies against EGFR (1:50), p-EGFR^Tyr1068^ (1:200), HIF-1α, and Notch1, Hes1 (1:400) were stained in sections of xenograft samples and EGFR (1:50), HIF-1α, and Hes1 (1:400) were stained in sections of *Tgfbr1/Pten* 2cKO tongue SCC samples by immunohistochemistry. The methods and processes were described as previously reported [20]. CD31 were stained in both xenograft and *Tgfbr1/Pten* 2cKO tongue SCC samples by frozen section immunohistochemistry. All slices were scanned using an Aperio ScanScope CS scanner with background substrate for each slice, and quantified using Aperio Quantification software (Version 9.1) for membrane, nuclear, or pixel quantification. Four random areas of interest were selected either in the epithelial or the cancerous area for scanning and quantification. Histoscore of membrane and nuclear staining was calculated as a percentage of different positive cells using the formula (3+)×3+(2+)×2+(1+)×1. Histoscore of pixel quantification was calculated as total intensity/total cell number. The threshold for scanning of different positive cells was set according to the standard vehicles provided by Aperio.

### Western blot analysis

Western blot were performed as previously described [22] with detail in Supplementary Material and Methods in S1 File.

### Statistical analysis

Graph Pad Prism version 5.00 for Windows (Graph-Pad Software Inc) was used for data analyses. Student *t* tests were performed to analyze the differences between two groups. Two-way ANOVA analysis was used for analyzing differences between animal treatment results. Two-tailed Pearson statistics were performed to correlate expression of EGFR with CD31, HIF-1α after confirmation of the sample with Gaussian distribution. All value was exhibited as Mean values ± SEM. *P*<0.05 were considered statistically significant.

## Results

### High EGFR expression in the *Tgfbr1/Pten* 2cKO mouse model of HNSCC

**Fig 1:**
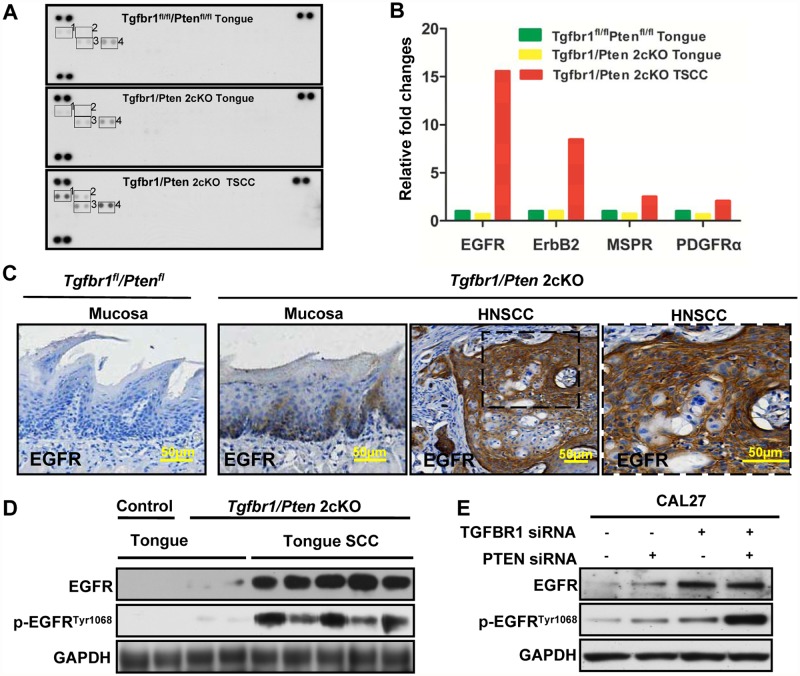
High EGFR expression in *Tgfbr1/Pten* 2cKO mouse HNSCC and *Tgfbr1* ^flox/flox^/ *Pten* ^flox/flox^ tongue. (A) Detection of RTK in 2cKO mice by antibody array. 1, EGFR; 2, ErbB2; 3, macrophage-stimulating protein receptor (MSPR); 4, platelet-derived growth factor receptor alpha (PDGFRα) (All groups n = 5). (B) Quantification of EGFR, ErbB2, MSPR, and PDGFRα expression. (C) EGFR expression was determined by immunohistochemistry in 2cKO HNSCC and *Tgfbr1* ^flox/flox^/ *Pten* ^flox/flox^ tongue; Scale bar, 50 μm. (D) EGFR and p-EGFR^Tyr1068^ expression levels were determined by Western blots in 2cKO mouse tongue, tongue SCC, and *Tgfbr1* ^flox/flox^/*Pten* ^flox/flox^ tongue, as well as in (E) CAL27 cells transfected with TGFBR1 siRNA and/or PTEN siRNA. GAPDH was detected on the same membrane and used as a loading control.

Tyrosine kinase dysregulation, overexpression and high activation are common phenomena in different cancers, including HNSCC. To examine the possible tyrosine kinase overexpression in the *Tgfbr1/Pten* 2cKO mouse model of HNSCC, we used a high-throughput antibody array with 39 RTKs to test the RTK expression of *Tgfbr1/Pten* 2cKO mouse tongue SCC in comparison with those of *Tgfbr1/Pten* 2cKO mouse tongue and *Tgfbr1* ^*flox/flox*^ */Pten* ^*flox/flox*^ tongue. Results revealed that the tyrosine kinases of EGFR, ErbB2, macrophage-stimulating protein receptor (MSPR) and platelet-derived growth factor receptor alpha (PDGFα) were highly expressed in *Tgfbr1/Pten* 2cKO mouse tongue SCC (Fig. 1A and 1B). Particularly, EGFR overexpression seemed to be the predominant molecular event in mouse tongue SCC (Fig. 1A and 1B). To confirm antibody array results, we used immunohistochemistry to directly observe the expression of EGFR in *Tgfbr1/Pten* 2cKO mouse tongue SCC. As shown in Fig. 1C, EGFR was almost negative in *Tgfbr1* ^*flox/flox*^ */Pten* ^*flox/flox*^ mucosa. The staining of EGFR in *Tgfbr1/Pten* 2cKO mouse HNSCC was even evidently stronger than that in in *Tgfbr1/Pten* 2cKO mouse mucosa (Fig. 1C). The results from western blots analysis (Fig. 1D) also validated this finding. More importantly, the activation of EGFR, p-EGFR^Tyr1068^ was much higher in *Tgfbr1/Pten* 2cKO mouse tongue SCC than that in the vehicle. Given that the mouse model was generated by conditionally knocking out *Tgfbr1/Pten*, we hypothesized that the expression levels of either EGFR or p-EGFR^Tyr1068^ increased after the knock down of TGFBR1 and PTEN, or both of them *in vitro*. The expression and activation of EGFR increased when the tongue cancer cells CAL27 were transfected with TGFBR1 and/or PTEN in siRNA (Fig. 1E). These results strongly indicate that tyrosine kinase dysregulation, particularly EGFR, is an important molecular event in the *Tgfbr1/Pten* 2cKO mouse model of HNSCC carcinogenesis and the deletion of *Tgfbr1* or *Pten* increased the expression and phosphorylation of total EGFR.

### Cetuximab treatment of CAL27 heterotopic xenograft tumors

**Fig 2:**
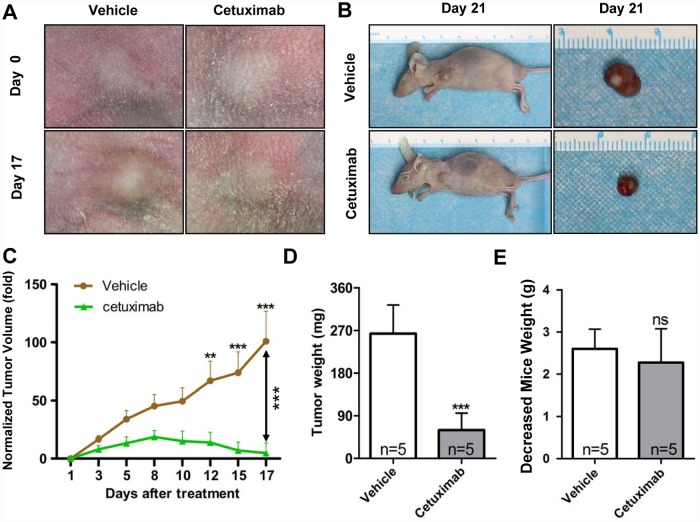
Cetuximab treatment of CAL27 xenografts tumor. (A) and (B) Tumor inhibition observed in CAL 27 xenograft treated with cetuximab (10mg/kg, i.p. twice per week, n = 5) or vehicle (PBS 100 μl, i.p. twice per week, n = 5). Examples of tumor inhibition in cetuximab-treated animals are photographed. (C) Tumor size from CAL 27 xenograft in both vehicle- and cetuximab-treated groups was assessed as indicated. Mean ± SEM; **, *P* ＜ 0.01; ***, *P* ＜ 0.001 versus the vehicle group; two-way ANOVA analysis. (D) Tumor dissected from each groups at the end point of assay was weighted. E Body weight of mice in each group was assessed twice per week. Mean ± SEM; ns, non significant; **, *P* ＜ 0.01; ***, *P* ＜ 0.001 versus the vehicle group; student *t* analysis

We treated heterotopic xenograft tumors derived from CAL27 cells with cetuximab to further identify the possible function of EGFR in HNSCC development. The mice received the treatment at 21 d post implantation and were euthanized for Western blot and immunohistochemical analyses on day 42. Cetuximab significantly delayed tumor growth (Fig. 2A and 2B). Fig. 2C showed the growth curves in tumors treated with cetuximab or vehicle, The mice administered with cetuximab showed partial tumor regression after 8 d of treatment., The cetuximab-treated mice showed significant tumor inhibition after 12 d of treatment (*P* < 0.01) compared with the vehicle-treated group. We harvested and weighted the tumor at end point of experiment and results revealed cetuximab possessed antitumor activity because the tumor in the vehicle-treated group had significantly higher weight than those in the cetuximab-treated group (Fig. 2D). The indicated dose of cetuximab exerted no significant toxicity to the mice because the mice weight between cetuximab- and vehicle-treated groups showed no significant difference (Fig. 2E). These results demonstrated that EGFR blockade effectively prevented tumor growth.

### Targeting EGFR by cetuximab delays HNSCC onset in *Tgfbr1/Pten* 2cKO mice

**Fig 3:**
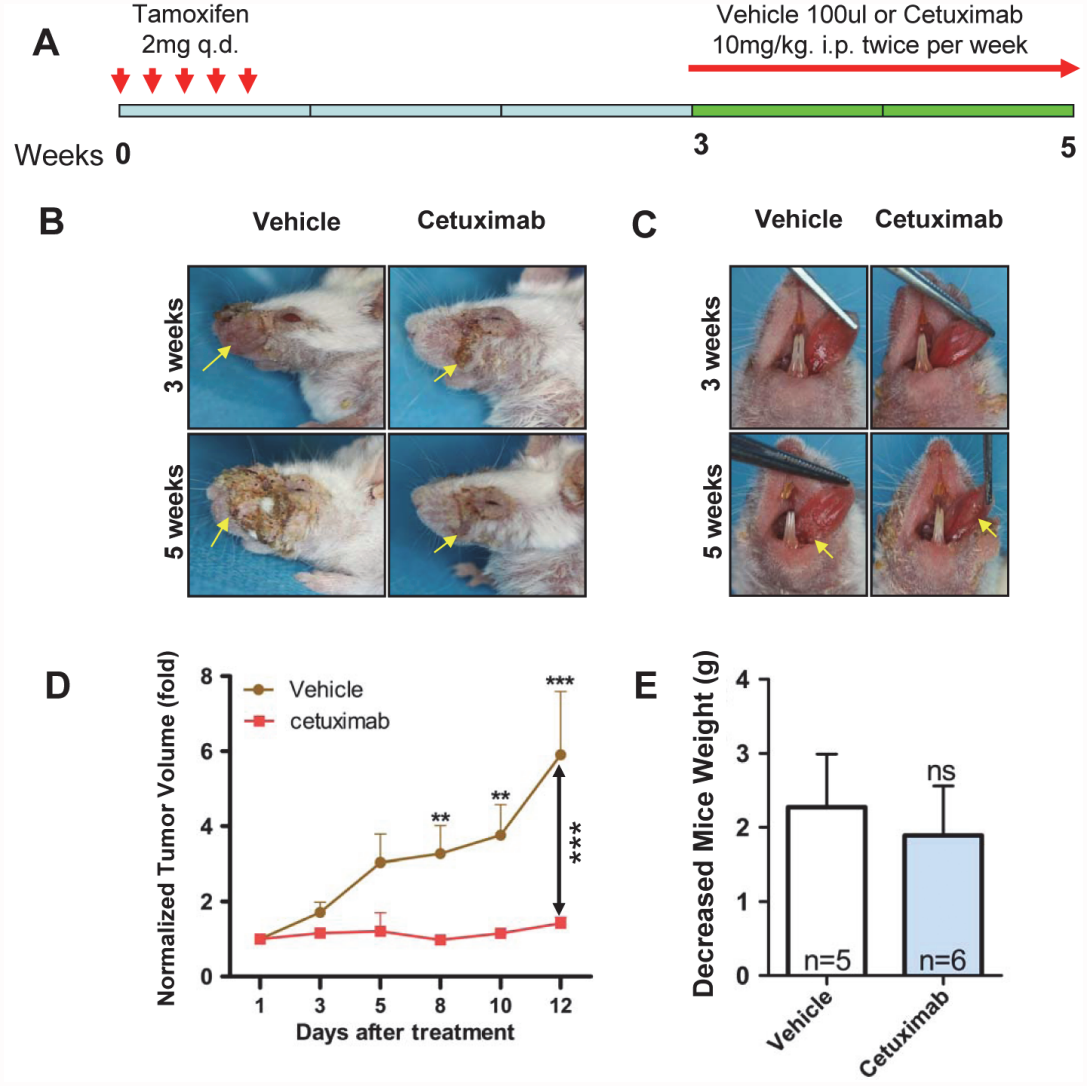
Targeting EGFR by cetuximab inhibit established HNSCC growth in *Tgfbr1/Pten* 2cKO mice. (A) A schematic showing the dosing schedule of cetuximab and vehicle to *Tgfbr1/Pten* 2cKO mice. (B) Representative mice with external head and neck (C) as well as tongue picture with cetuximab and vehicle treatment. (D) Tumor size from *Tgfbr1/Pten* 2cKO mice HNSCC in both vehicle- and cetuximab-treated groups was assessed as indicated. Mean ± SEM; **, *P* ＜ 0.01; ***, *P* ＜ 0.001 versus the vehicle group; two-way ANOVA analysis. (E) Body weight of *Tgfbr1/Pten* 2cKO mice in each group was assessed twice per week. Mean ± SEM; ns, non significant; student *t* analysis.

We performed a chemopreventive study on *Tgfbr1/Pten* 2cKO mice to determine whether or not an increase in EGFR was an early event in HNSCC tumorigenesis. We induced the onset of HNSCC tumor in *Tgfbr1/Pten* 2cKO mice as previously described [20]. The induction and drug administration strategies were shown Fig. 3A. Two weeks after the last tamoxifen oral gavages, the mice were treated with EGFR inhibitor or vehicle for 2 weeks. Cetuximab significantly (*P* < 0.001, n = 6) delayed tumorigenesis in external head and neck (Fig. 3B with quantification in Fig. 3D) and oral tongue tumors (Fig. 3C) in *Tgfbr1/Pten* 2cKO mice as compared with the vehicle group (n = 5). No significant weight loss was observed, indicating that cetuximab exerted no significant toxicity to these immuno-sufficient mice (Fig. 3E). These data indicated that EGFR blockade by cetuximab delayed the onset of HNSCC in 2cKO mice.

### Cetuximab inhibits tumor-induced angiogenesis *in vitro* and *in vivo*

**Fig 4:**
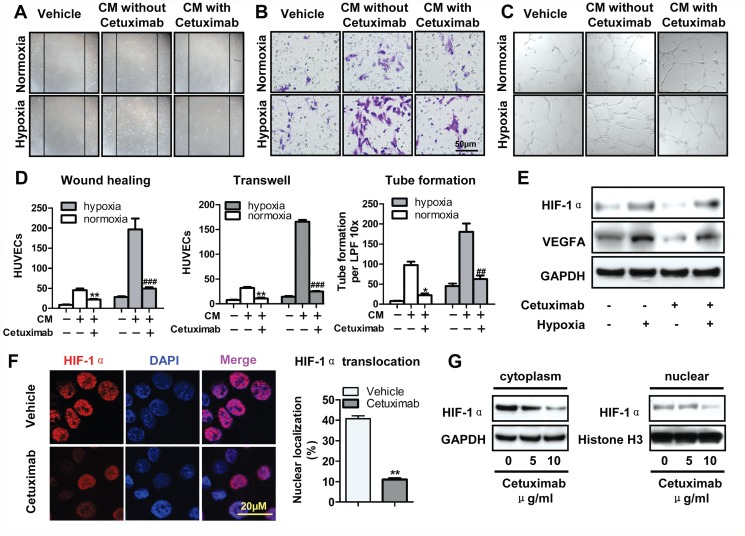
Cetuximab inhibits tumor angiogenesis *in vitro*. To collect conditional medium, CAL 27 cells were treated with or without the indicated cetuximab for 12 h, then cultured in fresh serum-deprived medium without cetuximab for another 24 h. Conditional medium-induced HUVECs migration assessed by *in vitro* wound-healing assay (Scale bars, 10μm) (A), (B) transwell assay (8μm pore size; Scale bars, 50μm) and (C) tube formation assay (Scale bars, 20μm). Vehicle means a blank medium was added; in the other two groups, as indicated, CM without or with cetuximab pretreatment was added. The hypoxia was 1% oxygen concentration. (D) Quantification of HUVECs migration and tube formation. Mean ± SEM; **, *P* ＜ 0.01 ***, *P* ＜ 0.001 versus the CM without cetuximab group in normoxia; ^##^, *P* ＜ 0.01; ^###^, *P* ＜ 0.001 versus the conditional medium without cetuximab group in hypoxia. (E) The expression levels of VEGFA and HIF-1α protein were analyzed by western blots after treated with hypoxia and cetuximab. (F) Cetuximab-treated (10μg/ml) reduced nuclear translocation of HIF1α in CAL27. Quantitative of nuclei translocation of HIF-1α in vehicle group and cetuxiamb-treated group from CAL 27 tumor tissues. Mean ± SEM, **, *P* < 0.01; student *t* analysis; Scale bars, 20μm. (G) The expression levels of HIF-1α protein in the cytoplasmic and nuclear extracts were analyzed by western blots after treated with hypoxia and cetuximab.

Digital pathology was performed to explore whether or not EGFR inhibition influences angiogenesis in 2cKO mice. Immunohistochemical staining showed that cetuximab downregulated EGFR, p-EGFR, and MVD in the xenograft tissues of CAL27 cells. Quantification of histoscore by using Aperio digital pathology validated the observation results. (S1A and S1B Fig.). The inhibition of EGFR expression and phosphorylation was also confirmed by Western blot (S1C Fig.). We collected the conditioned medium (CM) after pretreating CAL27 cells with cetuximab.We performed an *in vitro* migration assay to further confirm the function of cetuximab in angiogenesis *in vitro*. As shown in Fig. 4A, the CM from cetuximab-pretreated CAL27 cells reduced HUVEC migration as compared with the vehicle medium. Similar results were obtained in the Boyden transwell migration assay and tube formation assay under both hypoxic and normoxic culture conditions (Fig. 4B and 4C). The findings exhibited that CM significantly decreased HUVEC migration and tube formation after cetuximab pretreatment under both normoxic and hypoxic conditions when compared with the negative vehicle (Fig. 4D). Hypoxic culture conditions increased HUVEC migration as compared with normoxic culture conditions. The protein expression of HIF-1α and VEGFA were validated by western blots. Cetuximab reduced HIF-1α expression in normoxia and down-regulated VEGFA even in hypoxic condition (Fig. 4E). We further confirmed that 24 h of treatment with 10 μg/ml cetuximab reduced HIF-1α nuclear translocation in CAL27 cells under hypoxic culture conditions (Fig. 4F). To further detect the expression of HIF-1α, the protein levels expression of HIF-1α in the cytoplasmic and nuclear extracts were examined. As shown in Fig. 4G, cetuximab reduced expression HIF-1α in the nucleus in a concentration-dependent manner as compared with those in cells treated with vehicle. Aligned with this observation, cetuximab significantly inhibited HNSCC angiogenesis, and reduced HIF-1α nuclear translocation may be involved in this phenomenon.

### Notch1 signaling pathway is involved in cetuximab-reduced angiogenesis *in vitro*

**Fig 5:**
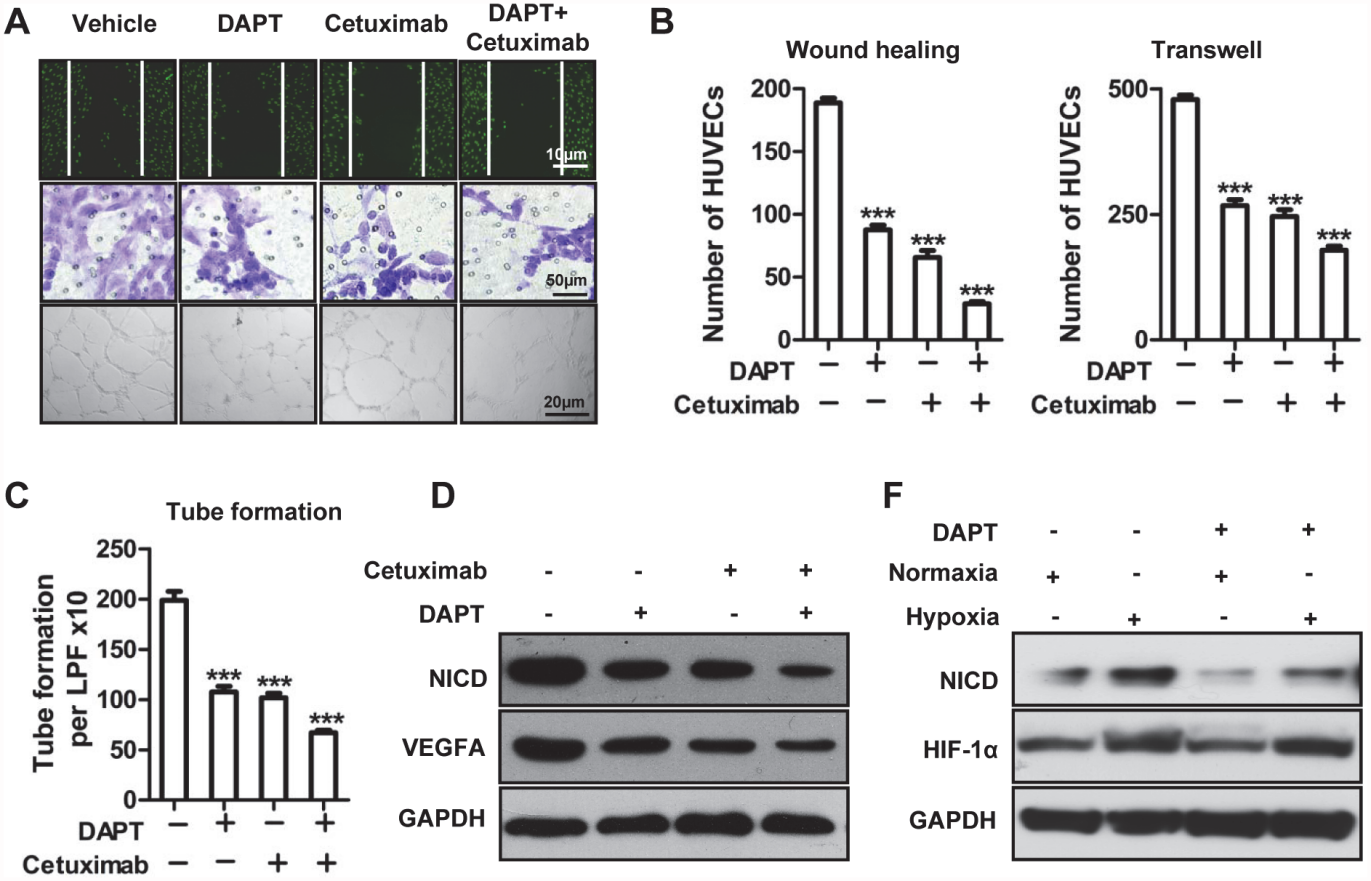
Notch1 signaling pathway is involved in cetuximab-reduced angiogenesis *in vitro*. (A) CM-induced HUVEC migration assessed by in vitro wound-healing, transwell (8 μm pore size; scale bars, 50 μm), and tube formation assays. (B) Quantification of HUVEC migration and (C) tube formation. Mean ± SEM; ***, *P* ＜ 0.001 vs. the vehicle group. (D) The expression levels of VEGFA and NICD proteins were analyzed by Western blot assay after treatment with DAPT, cetuximab, and the combination of DAPT and cetuximab. (F) The expression levels of HIF-1α and NICD protein were analyzed by Western blot assay with or without DAPT pretreatment under normoxic or hypoxic condition.

To further confirm whether Notch1 signaling pathway was involved in the preventive effect of cetuximab on tumor-induced angiogenesis, endothelial function assays were performed in the presence of DAPT, a widely used inhibitor for Notch1. As shown in Fig. 5A to 5C, the CM from cetuximab- or DAPT-pretreated CAL27 cells reduced HUVEC migration as compared with the vehicle medium using wound healing assays. HUVECs migration even were further inhibited when treated with the CM from Cetuximab combined with DAPT. Similar results were obtained in the Boyden transwell migration assays and tube formation assays (Fig. 5A to 5C). We next detected the expression of NICD, a cleaved fragment that transduced activated signals of Notch1, and VEGFA by western blots (Fig. 5D). The results showed that the DAPT or cetuximab reduced the expression of NICD as well as VEGFA. More, cetuximab further reduced the expression of VEGFA even in the presence of DAPT, may suggesting other downstream molecule moderated VEGFA either. To explore the interaction between HIF-1α and Notch1, protein levels of HIF-1α and NICD were tested by western blots. And we found hypoxia up-regulated the activation of Notch1 consistent with the up-regulation of HIF-1α, while DAPT showed no effect on HIF-1α in hypoxia (Fig. 5F), suggesting HIF-1α might play as upstream of Notch1 at least in CAL27 cell lines.

### Increased EGFR expression is related to HIF-1α and MVD in human HNSCC tissue

**Fig 6:**
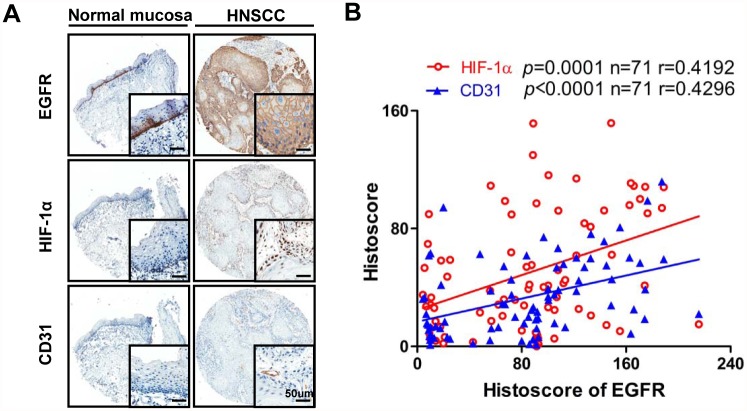
Increased expression of EGFR is related to increase of HIF-1α and CD31 in human HNSCC tissue. (A) Representative cores stain with EGFR, HIF-1α and CD31 in human normal mucosa (left) and HNSCC (right). (B) Positive correlation between the expression of EGFR, CD31 and HIF-1α in human normal mucosa and HNSCC tissue (n = 71, *P*＜0.0001; *P* = 0.0001 respectively). Aperio quantification software was used for histoscore, graph pad prism 5 for results analysis. Two-tailed pearson correlation statistics. Scale bars, 50μm.

We next evaluated the immunoreactivity of EGFR to HIF-1α and CD31 in human tissue array to further assess the correlation of EGFR with HIF-1α and MVD in human HNSCC. Of 54 cases, 48 presented positive membrane staining in almost all epithelial tumor areas of HNSCC tissue; only 10% of the mucosa core showed staining, and this staining was limited in the basal layer (Fig. 6A). Hypoxia is a common phenomenon in HNSCC. Intense HIF-1α nuclear staining was observed in a large proportion of tumor cells, suggesting hypoxia is a common phenomenon in HNSCC. The. staining of HIF-1α was considerably strong in invasive cancer. Most human HNSCC lesions were also highly angiogenic, as reflected by the strong staining of the vascular endothelial marker CD31 (Fig. 6A). EGFR expression positively correlated with high expression levels of HIF-1α (*P* = 0.0001, r = 0.4192) and CD31 (*P* < 0.0001, r = 0.4296) (Fig. 6B; statistic including normal mucosa and HNSCC, n = 71). These results further confirmed that increased EGFR expression was significantly associated with hypoxia and angiogenesis in HNSCC

### Cetuximab inhibits tumor-induced angiogenesis by downregulating HIF-1α and Notch1

We also examined the correlation of EGFR with Notch, another putative angiogenic molecule. Immunohistochemical staining showed that cetuximab treatment significantly reduced HIF-1α, Notch1, and Hes1 (putative downstream target of Notch1) (S2A and S2B Fig. *P* < 0.001 in each marker, n = 5 in each group) in CAL27 heterotopic xenogragft tumor. Supporting this result, the results from western blots showed that the protein levels of HIF-1α, Notch1, Hes1 and VEGFA were downregulated in CAL27 heterotopic xenograft tumor (S2C Fig. n = 3 for each group).

**Fig 7:**
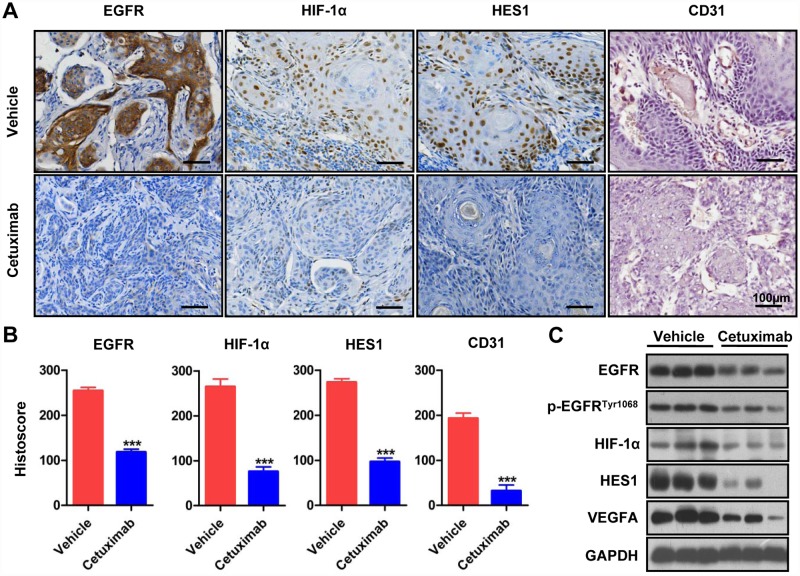
Cetuximab inhibit tumor-induction angiogenesis by down-regulating NOTCH1, HIF-1α pathway in *Tgfbr1/Pten* 2cKO mice HNSCC. (A) immunohistochemical analyses of EGFR, Hes1, HIF-1α and frozen section immunohistochemical analysis of CD31 in both cetuximab and vehicle-treated *Tgfbr1/Pten* 2cKO mice HNSCC tissues. (B) Quantitative of histoscore of EGFR, HIF-1α, HES1, CD31 expression in vehicle group and cetuximab-treated group from *Tgfbr1/Pten* 2cKO mice HNSCC tissues. Mean ± SEM, ***, *P* < 0.001; student *t* analysis; Scale bars, 100μm. (C) The expression levels of EGFR, p-EGFR^Tyr1068^, HIF-1α, HES1 and VEGFA protein were analyzed by western blots in both cetuximab and vehicle-treated *Tgfbr1/Pten* 2cKO mice HNSCC tissues.

Similar results were observed in 2cKO mouse HNSCC tissues, which are angiogenic and mimic human HNSCC in histological and molecule-expression patterns. Compared with the vehicle group (n = 7 from 5 mice), the residual cetuximab-treated HNSCC (n = 9 from 6 mice) showed downregulated HIF-1α, Hes1, EGFR, and CD31 expression (*P* < 0.001, Fig. 7A with quantification in Fig. 7B). The inhibition of EGFR expression and activation, HIF-1α, Hes1, VEGFA were also confirmed by western blots (Fig. 7C). These data further demonstrated that cetuximab downregulated tumor-induced angiogenesis in the 2cKO mouse model of HNSCC by inhibiting the HIF-1α and Notch1 pathways.

## Discussion

Understanding the molecular mechanisms underlying HNSCC initiation and tumor evolution is important to delay tumor progression. Among the signaling events in HNSCC, the persistent overexpression and activation of EGFR have emerged as putative drug targets for HNSCC treatment in preclinical and clinical investigations [23–25]. EGFR inhibitors, including cetuximab and lapatinab, can dramatically reduce tumor burden in HNSCC animal models [26] or patients [11] In the present study, the EGFR pathway is frequently activated in *Tgfbr1/Pten* 2cKO mice. EGFR overexpression may be related with Tgfbr1 and Pten downregulation. We assessed EGFR inhibition and angiogenesis in xenograft and transgenic mouse models of HNSCC. Results showed that EGFR inhibition with cetuximab can reduce tumor growth and angiogenesis in HNSCC.

Stroma and immune cells serve important functions in tumor angiogenesis [27]. Thus, the implantation of human HNSCC cells in immunodeficient mice may not completely reflect the clinical situation and may not accurately evaluate the efficacy of the drug on HNSCC angiogenesis [28]. *Tgfbr1/Pten* 2cKO mice are characterized by 100% penetrance; in addition, they mimic human HNSCC with similar morphology and molecular alteration. Therefore, we analyzed the effect of EGFR on angiogenesis using this mouse model. Results showed that EGFR inhibitors at clinically relevant doses can reduce the regulation of HIF-1α and Notch1 in this tumor type with limited side effects. This phenomenon resulted in reduced angiogenesis and tumor shrinkage.

In previous studies, we proved that the angiogenesis in 2cKO mouse HNSCC is related to HIF-1α activation by miR-135b [19]. Herein, the blockade of EGFR in this experiment rapidly decreased HIF-1α, a hypoxic biomarker frequently observed in advanced-stage HNSCC [29]. This effect likely involves the impact of cetuximab on angiogenesis by reducing HIF-1α nuclear translocation and/or reducing migration and chemoattractants, such as vascular endothelial growth factor A (VEGFA), for endothelial cells. This phenomenon prevents angiogenic signaling. The Notch signaling pathway is involved in the regulation of stem cell and neuronal cell death [30, 31]. However, recent evidence has shown that the Notch signaling pathway serves an important function during blood vessel formation and remodeling [32]. The Notch signaling pathway is involved in endothelial cell biology; it influences the budding of endothelial tip cells during angiogenesis initiation [33]. Notch1 was confirmed to be regulated by HIF-1α in a culture cell system [34]. Notch blockade can abolish the tumor resistance of glioblastoma to VEGF inhibitors [35, 36]. Blocking both Dll4/Notch and VEGF pathways synergistically inhibits tumor growth, which indicates the potential application of Notch inhibitors as new adjuvant chemotherapy reagents [37]. Dll4/Notch transcription was activated by Erk and PI3K signaling pathways, which were also downstream of canonical EGFR transduction [38]. Notch1 downregulation also reduced VEGF expression [39]. Thus, we hypothesized that cetuximab can decrease VEGF production and reduce HNSCC tumor angiogenesis by inhibiting the Notch signaling pathway. The present results showed that cetuximab inhibited the Notch1 signaling pathway by decreasing Notch1, Hes1, and VEGF expression in both nude mouse xenograft and 2cKO mouse models. Although these possibilities remain to be proven, the present findings support a unique anti-angiogenic function of cetuximab. That is, cetuximab can exert its antitumor activity by decreasing primary tumor growth and size, reducing HIF-1α instability, preventing endothelial cell initiation and migration, and downregulating VEGFA. These phenomena lead to the prevention of HNSCC angiogenesis.

High HIF-1α expression in HNSCC tissue is an important factor that predicts poor prognosis and resistance to chemotherapy and/or radiotherapy. The clinical application of EGFR as a molecular target of HNSCC therapy is a revolutionary event. However, the radiosensitization mechanism of cetuximab, a new adjuvant chemo-radiotherapy of HNSCC, still warrants further investigation. The emerging preclinical and clinical information about the promising beneficial angiogenetic effects of cetuximab on HNSCCs and our present findings on the capacity of cetuximab to downregulate Notch1 and HIF-1α signaling benefit HNSCC therapy. We can envision that the present study and prior reports may provide a rationale for the future clinical evaluation of cetuximab in an adjuvant setting, as a part of a molecular-targeted strategy after definitive treatment.

## Supporting Information

S1 File Supplementary Material and Methods. (PDF)

S1 Fig A. immunohistochemical analyses of the indicated biomarkers in both cetuximab and vehicle-treated CAL 27 tumor tissues. B. quantitative of histoscore of EGFR, p-EGFR, CD31 expression in vehicle group and cetuxiamb-treated group from CAL 27 tumor tissues. C. expression of EGFR, p-EGFR, CD31 was assessed by Western blotting. GAPDH was detected on the same membrane and used as a loading control. Mean ± SEM, ***, *P* < 0.001; student *t* analysis. Scale bars, 50μm.
(PDF)

S2 Fig A. immunohistochemical analyses of the indicated biomarkers in both cetuximab and vehicle-treated CAL 27 tumor tissues. B. Quantitative of histoscore of HIF1α, NOTCH1, HES1 expression in vehicle group and cetuxiamb-treated group from CAL 27 tumor tissues. Mean ± SEM, ***, *P* < 0.001; student *t* analysis; Scale bars, 50μm. C. The expression of HIF-1α, NOTCH1, HES1, and VEGFA were assessed by Western blotting. GAPDH was detected on the same membrane and used as a loading control.
(PDF)
