# Supplementary material for: Epidermal Growth Factor Receptor Inhibition Reduces Angiogenesis via Hypoxia-Inducible Factor-1α and Notch1 in Head Neck Squamous Cell Carcinoma
Source: PLoS One. 2015 Feb 27;10(2):e0119723. doi: 10.1371/journal.pone.0119723 (PMC4344331; doi:10.1371/journal.pone.0119723)
Supplement: S1 File — (PDF) [file pone.0119723.s001.pdf]

Supplementary Material

Epidermal growth factor receptor inhibition reduces angiogenesis via hypoxia-inducible factor-1α and Notch1 in head neck squamous cell carcinoma

Wei-Ming Wang [1, 2], Zhi-Li Zhao[1, 2], Si-Rui Ma[1, 2], Guang-Tao Yu[1], Bing Liu[2], Lu Zhang[1], Wen-Feng Zhang[2], Ashok B.Kulkarni[3], Zhi-Jun Sun[1, 2,*], Yi-Fang Zhao[1, 2,*]

Authors' Affiliations:

[1]The State Key Laboratory Breeding Base of Basic Science of Stomatology & Key Laboratory of Oral Biomedicine Ministry of Education, [2]Department of Oral and Maxillofacial-Head and Neck Oncology, School and Hospital of Stomatology, Wuhan University, Wuhan, 430079, China [3]Functional Genomics Section, Laboratory of Cell and Developmental Biology, National Institute of Dental and Craniofacial Research, National Institutes of Health, Bethesda, MD 20892, USA

[*]Corresponding Author: Zhi-Jun Sun or Yi-Fang Zhao, The State Key Laboratory Breeding Base of Basic Science of Stomatology & Key Laboratory of Oral Biomedicine Ministry of Education, Department of Oral and Maxillofacial-Head and Neck Oncology, School and Hospital of Stomatology, Wuhan University, Wuhan, China. Phone: 86-27-87686108; Fax: 86-27-87873260; E-mail: To Zhi-Jun Sun: zhijundejia@163.com, or to Yi-Fang Zhao: yifang@whu.edu.cn

Supplementary Material and Methods

Cell culture and conditional medium collection

The CAL27 cell line was obtained from the American type culture collection (ATCC,VA,USA) and maintained in Dulbecco's modified eagle medium (DMEM) supplemented with 10% FBS, in a humidified atmosphere of 95% air / 5% CO_2 at 37°C. When CAL27 cells were grown to 80% confluence after overnight incubation, cells were serum-deprived for 12 h and then treated with or without 10ug/ml cetuximab in serum-deprived DMEM for a indicated time(12h) in Anoxomat chambers (Mart Microbiology, Lichtenvoorde, the Netherlands) with appropriate oxygen concentrations for hypoxia(1% O_2) or normoxia (21% O_2). The cells were continue grow in serum-deprived endothelial basic medium (EBM, Lonza, Walkersville, MD, USA) medium for another 24h, and then the cleared supernatants were collected as conditional medium (CM) and stored at-80°C. Pooled human umbilical vein endothelial cells (HUVECs) were purchase from Lonza. HUVECs were grew in endothelial growth medium 2 (EGM-2, Lonza) and passage 3 to 8 were used in experiment, as previously described [1].

Wound-healing assay, Boyden chamber migration assay and tube formation

HUVECs cells were suspended in six-well culture plates, and grown to 90% confluence. A scratch was made across the cell layer using a 20μl sterile pipette tip. Then cells were added the CM. After 12 h, the cells were fixed and stained with acridine orange. Then the plates were observed under microscope and cells migrated into the gap were counted. Transwell Boyden chamber (Corning Life Sciences) were used to measure migration of endothelial cells. Transwell chamber contained a 6.5-mm-diameter polycarbonate filter (8μm pore size). Starved HUVECs cells (5×10^4 cells/well) incubated in 100μl of ECM were placed in the upper wells, whereas CM, as a chemo-attractant, was added to the lower wells. Then cells incubated for 12 h at 37°C. Using a cotton swab, HUVECs on the upper surface were carefully scrubbed off, and cells adhered to the lower membrane were fixed with 4% formaldehyde, stained by crystal violet, and observed under microscope. For capillary-like tube formation assay, HUVECs were seeded into 6-cm culture dishes coated with Matrigel (BD Biosciences, San Jose, CA) in CM. then the cell was cultured for 24 h at 37°C.Cells were fixed and observed under microscope. The formation of capillary-like

structures was counted.

Cell immunofluoresence and confocal microscopy

Immunofluorescence was performed as previous described [1]. When CAL 27 cells were grown to 80% confluence after overnight incubation, they were incubated in serum-deprived DMEM with or without cetuximab under hypoxic condition. After 12 h, cells were fixed with 4% formaldehyde in PBS (pH 8.0) for 10 minutes. After washed with PBS three times, cells were permeabilized for 5 min with 0.5% Triton X-100 in PBS and washed thoroughly three times with PBS, and 10% FCS in PBS were used to block nonspecific binding for 60 min, then cells were incubated with HIF1α antibody (1:50) at 4°C overnight. Then cells were washed thoroughly again three times with PBS. Primary antibodies were detected by incubation with goat anti-rabbit antibody IgG Dylight 594 (1:200) at room temperature for 60 min and cells were washed three times with PBS. Following DAPI staining nuclei, cells were token photos and analyzed by microscopy (CLSM-310, Zeiss).

Western blot analysis

Western blot were performed as previously described [2]. Briefly, CAL27 cells were treated with the indicated concentrations of cetuximab in DMEM containing 2% FBS for 24 h. Then the cells were lysed, and the total protein was separated using 10% SDS-polyacrylamide gel electrophoresis and transferred onto polyvinylidene fluoride membranes (Millipore Corporation, Billerica, MA). The immunoblots were cultured overnight at 4°C with the corresponding primary antibodies in blocking solution, followed by incubation with horseradish peroxidase-conjugated secondary antibody (Pierce Chemical, Rockford, IL). Then blots were developed by a West Pico ECL kit.

Supplementary references

1. Zhang L, Sun ZJ, Bian Y, Kulkarni AB (2013) MicroRNA-135b acts as a tumor promoter by targeting the hypoxia-inducible factor pathway in genetically defined mouse model of head and neck squamous cell carcinoma. Cancer Letters 331: 230-238.
2. Sun ZJ, Zhang L, Hall B, Bian Y, Gutkind JS, et al. (2012) Chemopreventive and chemotherapeutic actions of mTOR inhibitor in genetically defined head and neck squamous cell carcinoma mouse model. Clin Cancer Res 18: 5304-5313.

Epidermal growth factor receptor inhibition reduces angiogenesis via hypoxia-inducible factor-1α and Notch1 in head neck squamous cell carcinoma

Wei-Ming Wang [1, 2], Zhi-Li Zhao[1, 2], Si-Rui Ma[1, 2], Guang-Tao Yu[1], Bing Liu[2], Lu Zhang[1], Wen-Feng Zhang[2], Ashok B.Kulkarni[3], Zhi-Jun Sun[1, 2,*], Yi-Fang Zhao[1, 2,*]

Authors' Affiliations:

[1]The State Key Laboratory Breeding Base of Basic Science of Stomatology & Key Laboratory of Oral Biomedicine Ministry of Education, [2]Department of Oral and Maxillofacial-Head and Neck Oncology, School and Hospital of Stomatology, Wuhan University, Wuhan, 430079, China [3]Functional Genomics Section, Laboratory of Cell and Developmental Biology, National Institute of Dental and Craniofacial Research, National Institutes of Health, Bethesda, MD 20892, USA

[*]Corresponding Author: Zhi-Jun Sun or Yi-Fang Zhao, The State Key Laboratory Breeding Base of Basic Science of Stomatology & Key Laboratory of Oral Biomedicine Ministry of Education, Department of Oral and Maxillofacial-Head and Neck Oncology, School and Hospital of Stomatology, Wuhan University, Wuhan, China. Phone: 86-27-87686108; Fax: 86-27-87873260; E-mail: To Zhi-Jun Sun: zhijundejia@163.com, or to Yi-Fang Zhao: yifang@whu.edu.cn
