# Supplementary figures and images for: Epidermal Growth Factor Receptor Inhibition Reduces Angiogenesis via Hypoxia-Inducible Factor-1α and Notch1 in Head Neck Squamous Cell Carcinoma
Source: PLoS One. 2015 Feb 27;10(2):e0119723. doi: 10.1371/journal.pone.0119723 (PMC4344331; doi:10.1371/journal.pone.0119723)

# Supplementary Figure S1

**A**

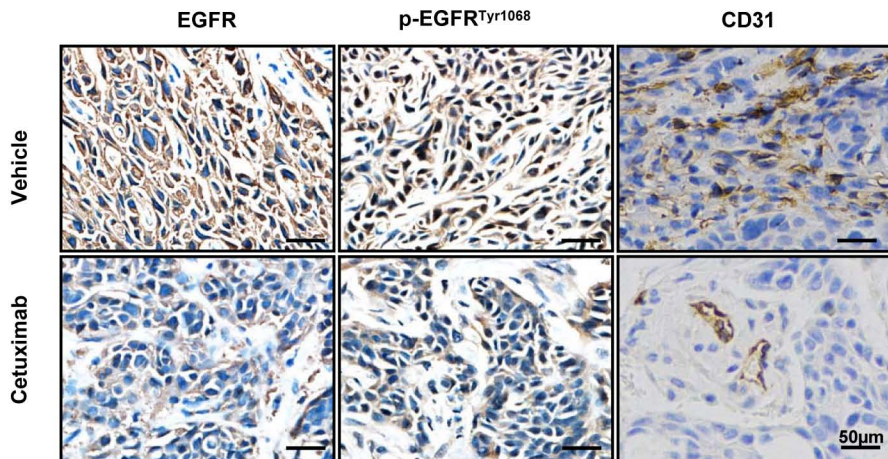

**B**

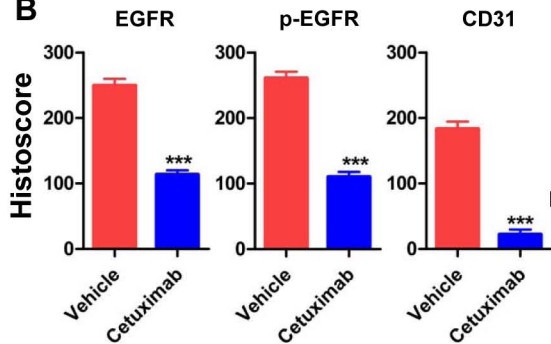

**C**

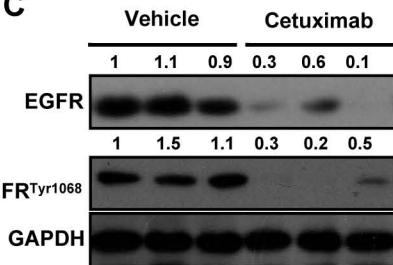

Supplement: S1 Fig — B. quantitative of histoscore of EGFR, p-EGFR, CD31 expression in vehicle group and cetuxiamb-treated group from CAL 27 tumor tissues. C. expression of EGFR, p-EGFR, CD31 was assessed by Western blotting. GAPDH was detected on the same membrane and used as a loading control. Mean ± SEM, ***, P < 0.001; student t analysis. Scale bars, 50μm. (PDF) [file pone.0119723.s002.pdf]

# Supplementary Figure S2

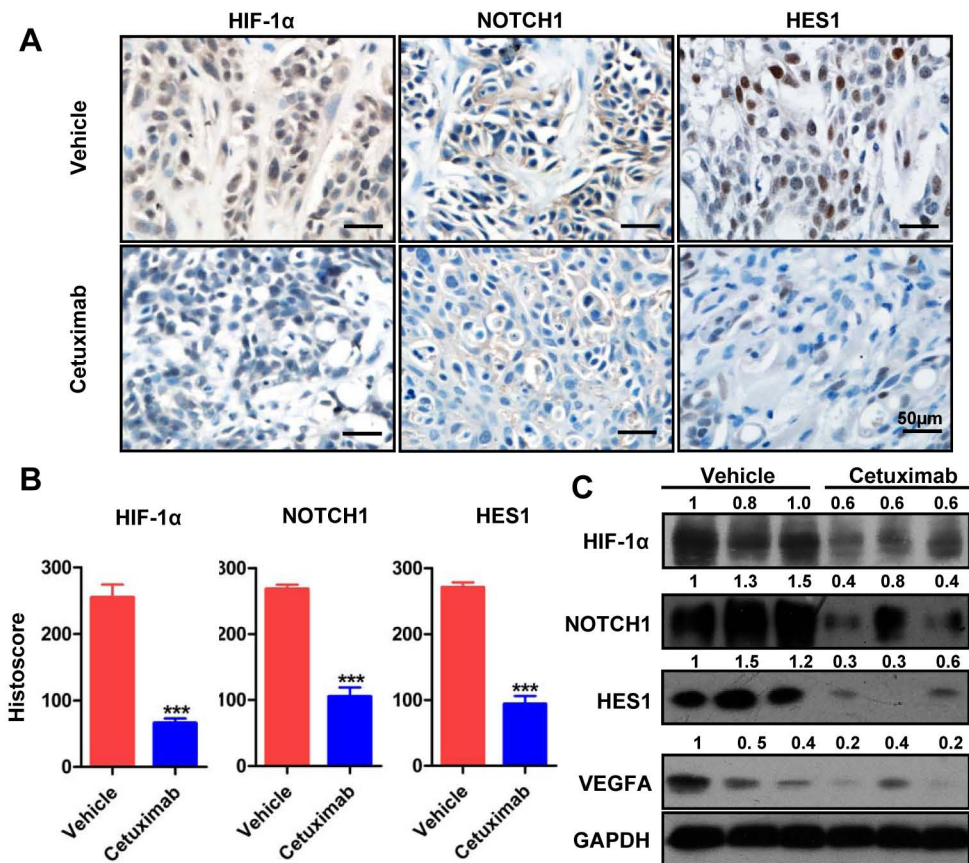

Supplement: S2 Fig — B. Quantitative of histoscore of HIF1α, NOTCH1, HES1 expression in vehicle group and cetuxiamb-treated group from CAL 27 tumor tissues. Mean ± SEM, ***, P < 0.001; student t analysis; Scale bars, 50μm. C. The expression of HIF-1α, NOTCH1, HES1, and VEGFA were assessed by Western blotting. GAPDH was detected on the same membrane and used as a loading control. (PDF) [file pone.0119723.s003.pdf]
